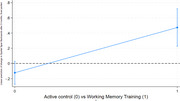# Computerized Working Memory Training vs Active Control in Mild Cognitive Impairment

**DOI:** 10.1002/alz70858_100914

**Published:** 2025-12-24

**Authors:** Trine Holt Edwin, Per R Nordnes, Anne‐Brita Knapskog, Gro CC Løhagen, Geir Ringstad, Haakon Hol, Petter E Emhjellen, Susanne MS Hernes

**Affiliations:** ^1^ University of Oslo, Oslo, Norway; ^2^ Oslo University Hospital, Oslo, Norway; ^3^ Sørlandet Hospital HF, Arendal, Arendal, Norway; ^4^ University of Bergen, Bergen, Bergen, Norway; ^5^ University of Oslo, Oslo, Oslo, Norway; ^6^ Oslo University Hospital, Oslo, Oslo, Norway

## Abstract

**Background:**

Working memory training may enhance cognitive function in Mild Cognitive Impairment (MCI). Given that one‐third of individuals with MCI progress to dementia within five years, interventions that delay progression are critical. This study investigates the effect of home‐based computerized working memory training on cognitive function in MCI.

**Method:**

In this multi‐center randomized controlled trial, participants with MCI were assigned to either a five‐week home‐based computerized working memory training program (*n* = 94) or an active control group playing web‐based solitaire (*n* = 53). All participants received weekly coaching by telephone. We analyzed the change in working memory, measured by the Spatial Span Backwards subtest (higher score indicates better working memory), from baseline to 3 months post‐intervention. A mixed‐effects model with robust standard errors was employed to account for both fixed and random effects. Fixed effects included treatment group, age, sex, and General Ability Index (GAI), while the random effect accounted for variability between centers.

**Result:**

Among 147 participants (mean age 68 years; standard deviation 7.5; 43% women), the Spatial Span Backwards score improved significantly in the working memory training group compared to the active control group at 3 months (least squares mean difference, 0.60; 95% confidence interval, 0.44 to 0.75 *p* <0.001).

**Conclusion:**

In individuals with MCI, home‐based computerized working memory training significantly improved working memory after 3 months compared to active controls.